# Deep Learning-Based Oral Cancer Detection Using Clinical Images

**DOI:** 10.7759/cureus.112795

**Published:** 2026-07-16

**Authors:** Arman Amin, Mark A Bachir, Akshay J Reddy, Ayush Vyas, Ashesh Ashesh, Jacob Webster, Rakesh Patel

**Affiliations:** 1 Biological Sciences, Florida State University, Tallahassee, USA; 2 Internal Medicine, California Northstate University College of Medicine, Elk Grove, USA; 3 Medicine, California University of Science and Medicine, Colton, USA; 4 College of Medicine, Northeast Ohio Medical University, Rootstown, USA; 5 Medicine, University of California Riverside School of Medicine, Riverside, USA; 6 Medicine, The University of Oklahoma, Norman, USA; 7 Internal Medicine, East Tennessee State University Quillen College of Medicine, Johnson City, USA

**Keywords:** clinical images, convolutional neural network, deep learning, efficientnet-b0, oral cancer

## Abstract

Oral cancer is a significant health concern where early detection greatly improves patient outcomes. This study develops and evaluates a deep learning model to automatically detect oral cancer from clinical photographic images. A convolutional neural network (CNN) was trained on a dataset of 750 oral lesion images sourced from Kaggle, using transfer learning with EfficientNet-B0 to compensate for limited sample size. The model's performance was validated on a held-out test set and assessed with accuracy, precision, recall, and receiver operating characteristic (ROC) curve analysis. Results indicate high diagnostic performance: the model achieved approximately 95% overall accuracy in distinguishing cancerous lesions from non-cancerous oral tissue. The CNN demonstrated a sensitivity of about 94% for identifying oral cancers and a specificity of about 95% for correctly recognizing non-cancer cases. The area under the ROC curve was 0.97, indicating excellent discriminative ability. Gradient-weighted Class Activation Mapping (Grad-CAM) visualizations were used as an exploratory interpretability method to identify image regions contributing to model predictions. These heatmaps may suggest overlap between model attention and visually relevant lesion regions, but they do not confirm that the model used clinically meaningful features. These findings suggest that deep learning could serve as a valuable tool in assisting dental professionals with early oral cancer detection, though further clinical validation is required before adoption.

## Introduction

Oral cancer, particularly oral squamous cell carcinoma (OSCC), poses a significant global public health burden. Worldwide, approximately 377,000 new cases of lip and oral cavity cancers are diagnosed annually, with over 177,000 deaths reported each year [1]. In the United States alone, the American Cancer Society estimated over 58,000 new cases of oral cavity and oropharyngeal cancer in 2023, making it one of the more prevalent head and neck malignancies [2]. Despite advances in treatment modalities, survival outcomes remain strongly influenced by stage at diagnosis. Late-stage detection continues to contribute to poor prognosis, while earlier diagnosis is associated with substantially improved treatment outcomes [3]. Systematic evidence further suggests that delays in diagnosis are associated with more advanced disease at presentation and poorer survival in head and neck cancers [4]. This underscores the importance of developing tools that can support earlier recognition of suspicious oral lesions.

Current diagnostic practice relies primarily on conventional oral examination (COE), which includes visual inspection and palpation of the oral mucosa during routine dental visits. Suspicious lesions are typically referred for biopsy and histopathological evaluation, which remains the gold standard for definitive diagnosis [3]. Diagnostic and referral delays remain important challenges in oral cancer care. Adjunctive image-analysis tools may help support future workflows by identifying images that warrant closer review, although such tools require validation in clinically representative datasets before use in practice [5]. In addition, oral leukoplakia and other potentially malignant disorders show variable clinical appearance and malignant transformation risk, making consistent recognition and risk assessment challenging [6]. A WHO-convened consensus has further highlighted the heterogeneity of oral potentially malignant disorders (OPMDs) and the need for standardized nomenclature and risk stratification to improve clinical recognition [7]. These diagnostic challenges can contribute to delayed referral, delayed biopsy, and diagnosis at more advanced stages.

Artificial intelligence (AI)-assisted pre-screening may help address this gap. A model that analyzes clinical photographs of the oral cavity could provide a rapid, objective, and reproducible assessment of suspicious lesions, especially in settings where specialist access is limited. Importantly, this type of system would not replace clinical judgment. Instead, it would function as a decision-support tool that helps dentists identify lesions that may require referral, biopsy, or further evaluation. Smartphone-based and photographic AI approaches have shown promise for oral cancer screening, suggesting that image-based deep learning systems may be practical for use in primary care, teledentistry, or community-based settings [8,9]. Mobile phone imaging has also demonstrated acceptable concordance with face-to-face oral examination by specialists, supporting the feasibility of remote photographic triage in low-resource environments [10].

Building on these efforts, deep learning models trained on clinical photographs have achieved expert-level classification of oral lesions and OSCC in retrospective and multi-center studies [11,12], and AI-assisted smartphone screening of OPMDs has further demonstrated practical utility for community-based programs [13]. Advancements in deep learning, particularly convolutional neural networks (CNNs), have shown substantial promise in medical image analysis by automatically extracting hierarchical image features and identifying complex patterns associated with disease [14,15]. CNN-based systems have demonstrated dermatologist-level performance in skin cancer classification, supporting the broader potential of deep learning for image-based clinical diagnosis [14]. Comprehensive surveys of medical image analysis have documented the rapid adoption of CNNs across imaging modalities and clinical specialties, including classification, detection, and segmentation tasks [16]. Transfer learning has also been shown to be useful in medical imaging because pre-trained CNNs can perform well when domain-specific datasets are limited, which is a common challenge in biomedical image classification [17]. Architectural advances such as EfficientNet, which uses a compound scaling strategy to balance network depth, width, and input resolution, have produced models that achieve state-of-the-art accuracy with substantially fewer parameters, making them well suited to medical image classification tasks with constrained computational resources [18].

In the dental and oral health domain, recent studies have applied deep learning to oral lesion classification, oral dysplasia prediction, and OSCC detection using clinical photographs, smartphone-based images, and confocal laser endomicroscopy images [8,9,19-22]. Notably, Jubair and colleagues developed a lightweight CNN based on a pre-trained EfficientNet-B0 backbone for binary classification of oral lesions from real-time clinical images, reporting an accuracy of 85% and an area under the receiver operating characteristic (ROC) curve (AUC) of 0.928, and demonstrating the feasibility of resource-efficient EfficientNet-based models for oral cancer screening [23]. However, direct comparison with the present study is limited because the datasets, image sources, acquisition conditions, class distributions, and case difficulty may differ. Therefore, this study is cited to support the feasibility of EfficientNet-based oral image classification, not to suggest that the present model is superior. A systematic review of AI applications in oral cancer further concluded that machine learning and deep learning approaches can analyze diverse oral image datasets with high accuracy and may meaningfully support early detection and prognostic assessment when integrated with clinical workflows [24]. Together, these studies suggest that CNN-based approaches can meaningfully assist in oral oncology by analyzing visual patterns that may be difficult to consistently detect through routine examination alone.

The present study focuses on developing a deep learning model for automated detection of oral cancer using clinical photographic images of oral lesions. We aimed to train and validate a CNN that can distinguish between images from patients with oral cancer and those from patients without oral cancer. Our approach leverages transfer learning with EfficientNet-B0 [18] to address the limited size of available oral image datasets. We evaluate the model's performance on a held-out test set of clinical images, report key metrics including accuracy, precision, recall, F1-score, and ROC-AUC, and examine visual explainability through Gradient-weighted Class Activation Mapping (Grad-CAM) heatmaps, which highlight the image regions most influential to model predictions and support clinical interpretability [25]. By emphasizing experimental performance and interpretability, this study seeks to demonstrate the feasibility of an AI-driven diagnostic aid and discuss its potential implications for dental practice.

## Materials and methods

Dataset and preprocessing

A dataset of clinical oral photographs was assembled for model training and evaluation from Kaggle. The collection included 500 images from patients with biopsy-confirmed oral cancer (malignant lesions) and 250 images from patients without oral cancer (healthy oral tissues or benign lesions), for a total of 750 images. Before feeding the data to the neural network, images were resized to a uniform resolution of 224×224 pixels and normalized for consistent pixel intensity ranges. Data augmentation techniques, such as rotations, flips, and brightness adjustments, were applied to the training images to increase effective sample diversity and help the model generalize better to new images. The dataset was obtained from the publicly available Kaggle dataset titled “ORAL CANCER DATASET” [26]. The dataset is organized into two source-provided categories: cancer and non-cancer. The premise of the source dataset is that the cancer-labeled images represent oral cancer cases, and these labels were used as provided by the Kaggle dataset; therefore, labels are described according to the source dataset classification. The dataset did not provide patient-level identifiers; therefore, the number of unique patients could not be determined. The train/validation/test split was performed at the image level. Because patient identifiers were unavailable, we could not confirm that images from the same patient were excluded across different splits.

Model architecture and training

We employed EfficientNet-B0 via transfer learning to leverage features learned from a large general image dataset [18]. The pre-trained EfficientNet-B0 model, initialized with ImageNet weights [27], was fine-tuned on the oral cancer dataset by replacing and retraining the final classification layers to output a binary prediction: oral cancer or no cancer. The model was trained using a supervised learning approach with binary cross-entropy as the loss function and the Adam optimizer to update model weights [28]. Training was conducted for a set number of epochs, and early stopping was implemented based on validation loss to prevent overfitting. During training, 10% of the dataset was set aside as a validation set to monitor the model's performance on unseen data and tune hyperparameters. All computational analyses were performed using Python 3 in Google Colaboratory. TensorFlow/Keras was used to implement EfficientNet-B0 and train the convolutional neural network [29,30]. Additional analyses and visualizations were performed using NumPy [31], scikit-learn [32], Matplotlib [33], and OpenCV [34]. The model was trained in Google Colaboratory using an available GPU runtime. The dataset was randomly split into training, validation, and test sets. The final held-out test set included 76 images. Images were resized to 224 × 224 pixels and normalized before model input. Data augmentation was applied to the training set and included rotations, flips, and brightness adjustments. The model was implemented in Python 3 using TensorFlow/Keras in Google Colaboratory. EfficientNet-B0 was initialized with ImageNet weights and used as the feature-extraction backbone. The final classification layers were replaced with a binary classification head. The model was trained using binary cross-entropy loss and the Adam optimizer. A classification threshold of 0.50 was used for binary prediction. A single train/validation/test split was used rather than repeated cross-validation.

Model evaluation

After training, the final model was evaluated on a separate test set that was not used in training or validation. Key performance metrics were calculated, including overall accuracy, precision, recall (sensitivity), specificity, and F1-score for both classes (oral cancer vs. no cancer). A confusion matrix was generated to provide a detailed breakdown of true positives, true negatives, false positives, and false negatives in the test results. We also plotted the ROC curve and calculated the AUC to assess the model's ability to discriminate between the two classes across varying decision thresholds. To interpret the model's decision-making, we applied Grad-CAM on a subset of test images [25]. Grad-CAM produced heatmaps highlighting the regions of each image that the CNN considered most indicative of the presence of cancer, offering insights into whether the model was focusing on clinically relevant areas of the lesions.

## Results

Model training

**Figure 1:**
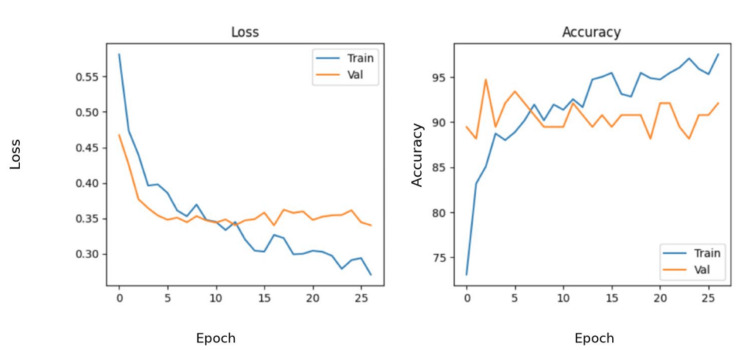
Training and Validation Loss and Accuracy Curves Training and validation loss (left) and accuracy (right) plotted across training epochs for the convolutional neural network (CNN). Both curves converge without significant divergence, indicating effective learning and minimal overfitting.

The CNN model converged effectively during training. Across successive epochs, the training loss steadily decreased while the training accuracy increased. Validation performance followed a similar trend without significant divergence from training performance, suggesting that overfitting was minimized. By the final epoch, the model achieved a high validation accuracy (approximately in the mid-90% range) and a low validation loss. The training and validation accuracy/loss curves are illustrated in Figure 1.

Test performance

**Figure 2:**
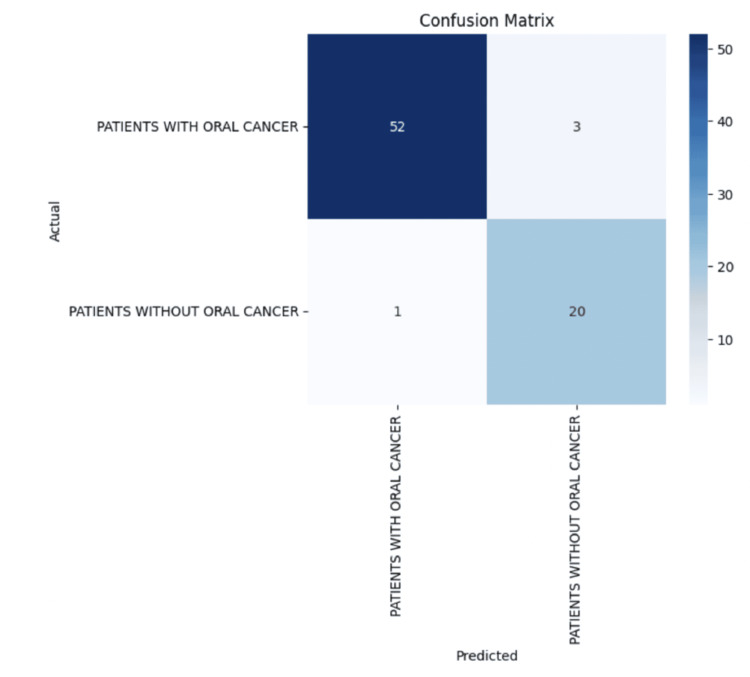
Confusion Matrix: Classification Outcomes on the Independent Test Dataset Confusion matrix displaying classification outcomes on the independent test dataset. Rows represent actual diagnoses, and columns represent model predictions. Values indicate the number of correctly and incorrectly classified cases.

On the independent test set of 76 images, including 55 cancer-labeled images and 21 non-cancer-labeled images, the model correctly classified 72 images. At the 0.50 classification threshold, the confusion matrix showed 52 true positives, 20 true negatives, one false positive, and three false negatives. The overall accuracy was 94.7%, sensitivity was 94.5%, specificity was 95.2%, positive predictive value (PPV) was 98.1%, and negative predictive value (NPV) was 87.0%. The ROC-AUC was 0.97. Because the test set was small, these estimates should be interpreted cautiously. The corresponding confusion matrix is shown in Figure 2.

ROC curve and AUC

**Figure 3:**
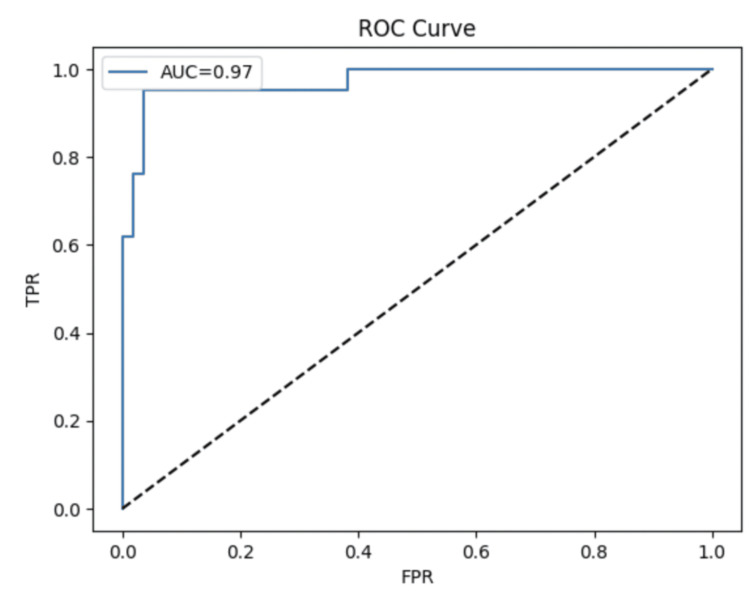
ROC Curve: Diagnostic Performance on the Test Dataset (AUC = 0.97) Receiver operating characteristic (ROC) curve demonstrating the convolutional neural network's diagnostic performance on the test dataset. The area under the curve (AUC) was 0.97.

The ROC analysis further confirmed the model's strong diagnostic performance. The ROC curve (Figure 3) rises sharply towards the upper left corner, indicating a high true positive rate alongside a low false positive rate across classification thresholds. The AUC was 0.97.

Grad-CAM visualizations

**Figure 4:**
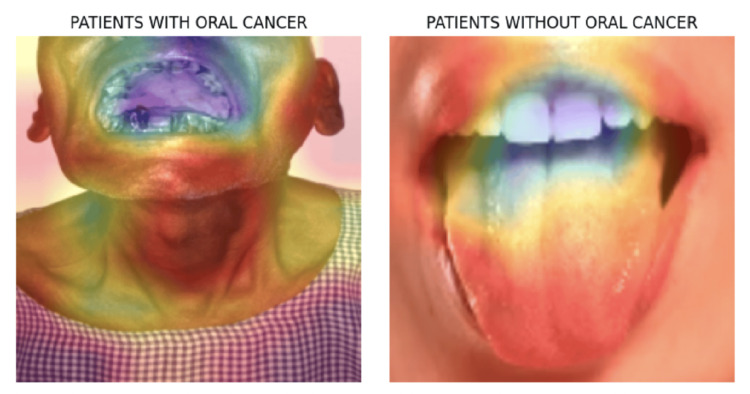
Grad-CAM Heatmaps: Regions Contributing to Convolutional Neural Network (CNN) Predictions in Representative Oral Lesion Images Gradient-weighted Class Activation Mapping (Grad-CAM) heatmaps illustrating regions contributing to the CNN's predictions in representative oral lesion images. Warmer colors indicate areas of greater influence on classification.

To explore the regions contributing to model predictions, Grad-CAM heatmaps were generated for representative test images (Figure 4). In images of oral cancer lesions, the visualizations typically highlighted the lesion area itself, concentrating on irregular mucosal areas or ulcerative regions that align with what a clinician would examine for malignancy. In images without cancer, the model's attention was more diffusely distributed across normal anatomical features.

## Discussion

This study demonstrates that a deep learning model can achieve high accuracy in detecting oral cancer from clinical photographic images of oral lesions. The CNN, trained on a relatively limited dataset using EfficientNet-B0 transfer learning, distinguished cancerous lesions from non-cancerous oral tissue with approximately 95% accuracy and an AUC of 0.97. These results are promising and broadly consistent with performance reported in other AI-assisted medical imaging studies. The high sensitivity (94%) is particularly clinically important, as it reflects the model's ability to identify the majority of true cancer cases, reducing the risk of missed diagnoses that could delay treatment. The high specificity (95%) simultaneously limits unnecessary referrals and patient anxiety associated with false positives.

Our findings align with and extend a growing body of literature demonstrating the utility of deep learning in dental and oral health applications. Aubreville et al. showed that CNNs could reliably distinguish OSCC from healthy tissue in confocal laser endomicroscopy images, achieving diagnostic accuracy comparable to expert clinicians [35]. Tanriver et al. similarly demonstrated CNN-based classification of common oral lesions from clinical photographs, reporting AUC values above 0.90 [19]. Our results are consistent with these findings and further validate the applicability of transfer learning - specifically EfficientNet-B0 - to this domain. The EfficientNet architecture's compound scaling approach provides an efficient balance between model depth, width, and resolution, making it well-suited for medical image classification tasks where dataset sizes are limited [18]. By initializing with pretrained ImageNet weights, the network leveraged general feature detectors and then specialized on the visual characteristics of oral lesions, rather than learning from scratch. The Grad-CAM results provide exploratory visualization of regions contributing to model predictions; however, these heatmaps should not be interpreted as proof that the model used clinically meaningful features.

The broader impact of this work lies in its potential for integration into real-world dental practice. A significant proportion of oral cancers are diagnosed at advanced stages due to delayed identification in primary care settings, where clinician experience with oral mucosal pathology varies considerably [3]. An AI pre-screening tool that analyzes a standard intraoral photograph could serve as a consistent, accessible second opinion - particularly valuable in community dental clinics, rural practices, or teledentistry platforms where specialist access is limited. The relatively straightforward image input (a clinical photograph) means such a system could be deployed via a smartphone interface or integrated into existing dental imaging workflows without requiring specialized equipment.

Despite the high performance, there are important limitations to acknowledge. The dataset, while adequate for a proof-of-concept investigation, may not capture the full variability of lesions encountered in a general patient population. Images sourced from Kaggle may reflect specific camera conditions, lighting environments, and patient demographics that differ from real-world clinical settings, potentially limiting the model's generalizability. Additionally, the class imbalance in the dataset (2:1 ratio of cancer to non-cancer images) may have influenced model learning, though the balanced sensitivity and specificity suggest this was adequately managed. A review of the misclassified cases reveals a pattern worth noting: the small number of false negatives (missed cancers) tended to involve images with suboptimal lighting, partially obscured lesion areas, or early-stage lesions with subtle visual characteristics. This suggests that image acquisition quality is an important factor in model reliability, and future work should incorporate standardized photographic protocols to minimize this source of variability. Strategies such as lowering the classification threshold to favor sensitivity, or deploying the model as a triage aid rather than a standalone diagnostic tool, could further mitigate the risk of missed diagnoses in a clinical setting.

Future work should prioritize external validation using independent patient cohorts and prospective clinical trial settings to determine whether the model’s performance generalizes beyond the source dataset. Expanding the task to multi-class classification, including differentiation of leukoplakia, erythroplakia, other potentially malignant oral disorders, and frank OSCC, would provide greater clinical granularity and utility. Larger and more diverse datasets, ideally collected across multiple clinical centers and representative of varied demographic populations, would further strengthen model robustness and generalizability. In addition, human factors research evaluating how dental professionals interpret, trust, and act upon AI-generated outputs will be essential for guiding safe and effective clinical integration. Although the source dataset is presented as an oral cancer dataset with cancer and non-cancer labels, individual patient-level histopathology reports were not available to the authors for independent verification. Therefore, the reported classification performance should be interpreted in the context of the dataset-provided labels. Future studies should use datasets with directly accessible clinical annotations, histopathologic confirmation, and patient-level metadata. Another important limitation is the absence of patient-level identifiers in the public dataset. As a result, the number of unique patients could not be determined, and a patient-disjoint split could not be confirmed. If multiple images from the same patient were present across the training and test sets, model performance may have been overestimated. Future work should therefore employ patient-level splitting using prospectively collected, clinically annotated datasets. Calibration analysis was not performed because raw predicted probabilities were not available for the current revision. Accordingly, this study evaluates discrimination and classification performance rather than probability calibration. The dataset had a 2:1 ratio of cancer-labeled to non-cancer-labeled images. No specific class-imbalance correction technique, such as class weighting, resampling, or threshold adjustment, was applied. Therefore, class imbalance remains a limitation and may have influenced model training and predictive values. Finally, some training hyperparameters, including the exact learning rate, batch size, random seed, and early stopping criteria, were not available for independent confirmation at the time of revision. Future studies should provide complete code, model configuration files, and training logs to improve transparency, reproducibility, and independent validation.

## Conclusions

We developed an EfficientNet-B0-based CNN capable of detecting oral cancer from clinical photographic images with approximately 95% accuracy and an AUC of 0.97. These findings suggest that deep learning may serve as a useful adjunct to COE by helping identify suspicious lesions that warrant further clinical evaluation, referral, or biopsy. The use of Grad-CAM visualizations provided an additional layer of interpretability by highlighting image regions that contributed to model predictions, which may help support clinician trust in AI-assisted decision-making. However, this model should be viewed as a decision-support tool rather than a replacement for clinical judgment or histopathologic diagnosis. Further external validation using larger, more diverse, and prospectively collected clinical datasets is needed before implementation in routine dental or oral medicine practice. Overall, this study supports the potential role of AI-enhanced image analysis in improving early oral cancer detection and guiding timely patient care.
